# Modulation of Physical and Thermal Properties in Wild Banana (*Musa balbisiana* Colla) Seed Powder by Moisture Variations

**DOI:** 10.1155/2024/8846365

**Published:** 2024-02-22

**Authors:** Murlidhar Meghwal, Chitra Lekhwar, Yogesh Kumar, Vivek Kumar, Rajat Suhag, Pramod K. Prabhakar

**Affiliations:** ^1^Department of Food Science and Technology, National Institute of Food Technology Entrepreneurships and Management, Sonipat 131028, Haryana, India; ^2^Department of Agricultural and Food Sciences, University of Bologna, Piazza Goidanich 60, 47521 Cesena, Italy; ^3^Faculty of Agricultural, Environmental and Food Sciences, Free University of Bolzano, Piazza Università, 1, Bolzano 39100, Italy

## Abstract

Engineering and flow properties of banana seed powder as a function of moisture content are important for processing, handling, packaging, and transport processes. The bulk density, tapped density, and porosity increased from 377.37 to 427.36 kg m^−3^, 622.08 to 746.33 kg m^−3^, and 38.99-43.74%, respectively, within the increasing moisture content range. The Hausner ratio (Hr) and Carr's compressibility index (CI) significantly (*p* < 0.05) increased with an increase in moisture content (6.16-19.56% db) of banana seed powder, whereas HR fell in the range of 1.4-2.0, indicating cohesive characteristics of banana seed powder. The angle of repose, angle of spatula, and angle of fall exhibited a linear increase, ranging from 40.6° to 49°, 33.4° to 39.4°, and 35.6° to 42.6°, respectively, with increasing moisture content. The static coefficient of friction was found to be highest for aluminium and glass surfaces and least for stainless steel. The water activity and swelling power of banana seed powder showed a significant increase, while the solubility and oil absorption capacity exhibited a significant decrease within the range of increasing moisture content. The thermal characteristics of wild banana seed powder, such as thermal conductivity (0.16 to 0.20 Wm^−1^ K^−1^) and volumetric specific heat (0.58 to 0.99 MJm^−3^ K^−1^), demonstrated an increasing trend as the moisture content increased. However, the thermal diffusivity showed a decrease from 0.31 to 0.19 (×10^−6^ m^2^s^−1^) with the increase in moisture content.

## 1. Introduction

India annually produces a staggering 29 million tonnes of bananas, making it one of the leading global producer [1]. *Musa balbisiana* Colla, commonly known as Bhimkol in certain regions of Assam, is a wild variety of banana found in evergreen forests [2]. *Musa balbisiana* exhibits resistance to drought and greater tolerance to dry conditions compared to other banana species. This is attributed to genetic variations in the banana's genome, which can be categorized as ABB or AAB [3]. *Musa balbisiana* displays a bluish-green color and is characterized by numerous small seeds in its raw form. This banana species has a wide distribution across India and serves as a reservoir for various phytochemicals, including flavonoids and polyphenols [4].

*Musa balbisiana* is a fruit abundant in nutritional value, encompassing carbohydrates, amino acids, vital vitamins (vitamin C and vitamin B6), and minerals (phosphorus, potassium, zinc, calcium, and manganese). With its exceptional nutrient profile, it serves as an excellent dietary choice, particularly as a wholesome option for baby food, promoting healthy weight gain in infants [5, 6]. Moreover, this fruit exhibits antioxidant potential and hypotesticular activity, acts as an antimicrobial agent, holds promise as an antidiabetic agent, and possesses anti-inflammatory properties [1, 4]. Bananas are the good source of starches and can also be modified into resistant forms, effectively slowing digestibility and reducing the glycemic index. The beverage, bakery, and confectionery industries have delved into exploring the utilization of banana starches for innovative formulations, capitalizing on their remarkable texturizing capabilities, mouthfeel enhancement, and palatability [7]. Additionally, Kaur et al. [8] mentioned that starch is common as biofiller both in native and nanolevels, making it the most suited route to achieve application in the human concerning fields of food, pharma, and textile industries.

The seeds of *Musa balbisiana* are nutritionally rich and have been found to contain ferulic acid, fatty acids (palmitic and stearic acid), and polyphenols. Notably, apiforol, a photoactive compound derived from these seeds, has demonstrated promising antidiabetic properties. Therefore, it holds potential as a medicinal option for diabetic patients, exhibiting inhibition of *α*-glucosidase [9]. According to a study, *Musa balbisiana* seeds, when combined with a fruit extract of *Terminalia chebula*, have been used to prepare a male contraceptive called “Contracept-TM” and the results confirm the traditional reputation of *Terminalia chebula* and *Musa balbisiana* as a male contraceptive [10].

Due to the potential benefits associated with wild banana seeds, converting them into powder form can enhance the bioavailability of the active ingredients and improve the absorption of the compounds. Wild banana seeds can be achieved in powdered form with the help of different types of mills such as hammer mills, rotor mills, jet mills, and ball mills. Furthermore, moisture content has been shown to influence the storage behavior of wild banana seeds/powder, indicating both orthodox and intermediate nature and, more recently, at the orthodox and intermediate classification threshold [11, 12].

The grinding of seeds is a process that demands significant energy, and its efficiency can be influenced by various physical properties and moisture content of banana seeds. Therefore, understanding the moisture-dependent properties of food materials is essential for process design, assessing other characteristics, ensuring quality, and facilitating the proper handling and packaging of any food material or agricultural produce [13, 14]. Thus, the present study is aimed at investigating moisture-dependent engineering properties of wild banana seed powder, such as powder volume, bulk density, tapped density, porosity, Hausner's ratio, Carr's index, angle of repose, angle of slides, angle of spatula, angle of fall, water activity, solubility, swelling power, oil absorption capacity, compressibility index, and thermal properties such as volumetric specific heat, thermal diffusivity, and thermal conductivity.

This study's significance lies in its detailed exploration of moisture-dependent engineering properties of wild banana seed powder, providing valuable insights for the food industry and consumers alike. For food manufacturers, this knowledge facilitates the development of high-quality products with optimized textures, extended shelf life, and efficient production processes, ultimately reducing costs and energy consumption. Additionally, it enables the creation of diverse, healthier food options tailored to consumer preferences. For consumers, this research ensures consistent quality, enhancing taste and texture experiences and promoting food safety by optimizing moisture levels. In summary, the study's findings bridge the gap between scientific understanding and practical applications, benefiting both the food industry and consumers through improved product quality, innovation, and safety assurance.

## 2. Materials and Methods

### 2.1. Sample Collection and Preparation

**Figure 1:**
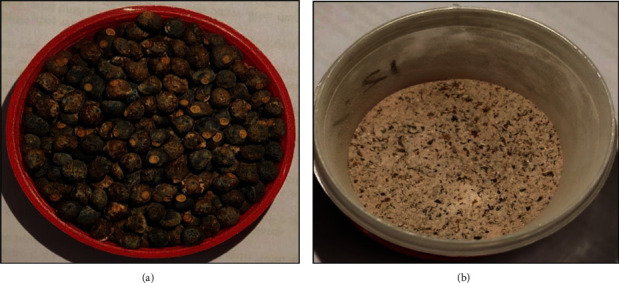
Pictorial view of (a) wild banana (*Musa balbisiana*) seeds and (b) banana seed powder.

The wild banana fruits were obtained from a farm at the National Institute of Food Technology Entrepreneurship and Management, Sonipat, Haryana, India (28° 52′ 41.6^″^ N 77° 08′ 01.7^″^ E) during the summer of 2023. Seeds were manually separated from the fruit, and the obtained seeds contained traces of pulp, so they were washed using distilled water. Furthermore, the seeds were dried in sunlight for 6-7 days. The moisture content was determined using the hot air oven (Macro Scientific Works Pvt. Ltd., RM-SP-325, India) at 105°C for 24 h [15]. The dried seeds (Figure 1(a)) contained moisture levels of 6.65% (db).

To achieve the desired moisture levels of 10, 14, 18, and 22% (db), the moisture level of the seeds was increased by adding distilled water. Subsequently, the seeds were packed and refrigerated at 4°C for 1 day to ensure even distribution of the water. Before starting the experiment, the required amount of seeds was taken out of the refrigerator and allowed to equilibrate at room temperature for 2 h [16–19]. The amount of distilled water required to reach the desired level of moisture was calculated using the following equation:

(1) $$\begin{array}{c} Q.D=Wbi\frac{\left(Mr-Mi\right)}{100-Mr}, \end{array}$$

where *Q*.*D* is the amount of distilled water added (g), Wbi is the initial mass of wild banana seeds (g), Mi is the initial moisture content of wild banana seeds (% (db)), and Mr is the required moisture content of wild banana seed (% (db)) [20].

Furthermore, banana seeds were ground using a hammer mill (Onyx Industries Pvt. Ltd., India) for 6-8 min, and the temperature of grounded powder (Figure 1(b)) ranged from 42 to 44°C. The hammer mill was equipped with a single-phase AC motor operating at 1 HP and 2800 rpm. The powder was sieved through a 200 *μ*m mesh size using a vibratory-type sieve shaker for 15 min. Grinding the seeds resulted in a decrease in moisture content within the obtained powder due to the elevated temperature of the mill during the grinding process. The powder's final moisture content (Mc) was measured at 6.16, 9.14, 13.12, 16.39, and 19.56% db, which were subsequently utilized for the study. The initial moisture value (6.16% db) was obtained without adding water, while the other four levels (9.14, 13.12, 16.39, and 19.56% db) were obtained by adding distilled water.

### 2.2. Volume, Bulk Density, Tapped Density, and Porosity of Porous Powder

Powder volume was determined as described by Santomaso et al. [21]. The seed powder was filled in a hollow graduated cylinder, and the height and inner diameter of the cylinder were noted. The powder volume was calculated using the following equation:

(2) $$\begin{array}{c} V=\pi \;R^{2}H, \end{array}$$

where *V* is the powder volume, *R* is the radius of the cylinder, and *H* is the height of the cylinder.

The bulk density of powder is the ratio of the weight of powder to the volume occupied by the powder and was expressed as kg/m^3^. The tapped density was calculated by tapping a filled measuring cylinder of 100 ml with ground banana seed powder until no further volume could be added; then, the sample was weighed [21, 22]. The powder bulk density and tapped density were determined using the following equations:

(3) $$\begin{array}{c} \rho_{\text{b}}=\left(\frac{W_{\text{p}}}{V_{\text{pp}}}\right), \end{array}$$

where *W*_p_ is the amount or weight of the poured powder (kg) and *V*_pp_ is the volume of the poured powder (m^3^).

(4) $$\begin{array}{c} \rho_{\text{t}}=\left(\frac{W_{\text{t}}}{V_{\text{tp}}}\right), \end{array}$$

where *W*_t_ is the weight of tapped powder (kg) and *V*_tp_ is the volume of the tapped powder (m^3^).

After measuring the bulk and tapped powder density, the porosity of the powder (*ε*, %) was measured using the following equation [20, 23]:

(5) $$\begin{array}{c} \varepsilon \left(\%\right)=\left[1-\left(\frac{\rho_{\text{b}}}{\rho_{\text{t}}}\right)\right]\times 100. \end{array}$$

### 2.3. Hausner's Ratio and Carr's Compressibility Index

Hausner's ratio (Hr) and Carr's compressibility index (CI) are important properties used to assess the flowability of the powder [24]. Hr was calculated as the ratio of tapped density to bulk density, while CI represent the powder ability to get compressed. Hr and CI were determined using

(6) $$\begin{array}{c} \text{Hr}=\left(\frac{\rho_{\text{t}}}{\rho_{\text{b}}}\right), \end{array}$$

(7) $$\begin{array}{c} \text{CI}=100*\left(1-\frac{1}{\text{Hr}}\right), \end{array}$$

where *ρ*_t_ is the tapped density of powder (kg/m^3^) and *ρ*_b_ is the bulk density of poured powder (kg/m^3^).

If Hr > 1.25 is the indication of poor flowability, Hr < 1.25 indicates that the powder is freely flowable.

### 2.4. Angle of Repose (*α*), Spatula, Fall, and Static Coefficient of Friction

#### 2.4.1. Angle of Repose (*α*)

The angle of repose was measured by using a hollow cylinder with a height of 5.0 cm and a diameter of 5.5 cm. The cylinder was placed on a circular plate, and wild banana seed powder was poured into it until it reached the rim. Slowly, the cylinder was removed, allowing the wild banana seed powder to settle and form a cone on the plate [11]. At the stationary condition diameter (cm), the height (cm) of the heap was noted and the angle of repose was determined using

(8) $$\begin{array}{c} \alpha =\tan-1\left(\frac{2H}{D}\right), \end{array}$$

where *α* is the angle of repose, *H* is the height of the cone (cm), and *D* is the diameter of disk (cm).

#### 2.4.2. Angle of Fall

To measure the angle of fall (*α*_f_), a weight of 15 g was dropped three times from a predetermined height of 9 cm. This action caused vibrations and forces that resulted in a change in the previously determined angle of repose of the powder. The new angle is known as the angle of inclination or angle of fall. In general, a smaller value of *α*_f_ indicates enhanced flowability, and it is expected to be lower than the angle of repose [25].

#### 2.4.3. Angle of Spatula

A flat-blade spatula, measuring 22 cm in width, was immersed parallel to the base of the container into the bulk powder. It was subsequently lifted directly and tapped gently, following which the angle of a tangent from the edge of the spatula was measured. Multiple measurements were taken, and the average values were recorded to obtain a reliable estimate of flowability. If the measured angle falls below 40°, it indicates that the material exhibits free-flowing characteristics [25].

#### 2.4.4. Static Coefficient of Friction

The coefficient of friction is a measure of the resistance to sliding between a surface and grains or food powders. To determine this coefficient, three distinct surfaces were chosen: aluminium, silica glass (*R*_a_ = 1 nm), and stainless steel (*R*_a_ = 0.48 *μ*m). These surfaces are commonly utilized for rolling powders and are relevant in the transportation and packaging of granular food materials. The surface was inclined using a screw until the structurally formed box of powder began to slide downwards [11, 13]. The angle of tilt was then recorded, and the coefficient of friction was then determined by

(9) $$\begin{array}{c} \mu =\tan\left(\text{ø}\right). \end{array}$$

### 2.5. Water Activity, Solubility, and Swelling Power

The water activity of banana seed powder was determined according to the method suggested by Fazaeli et al. [26]. Water activity (*a*_w_) measures how tightly water is bounded within a substance. It was analyzed using AquaLab Pre Works at Niftem, Kundli, by taking 15 g of sample in the sample holder. The reading was taken at 25°C with 5 independent replications.

Solubility and swelling power were determined according to the method used by Cano-Chauca et al. [27] and Tamuno [28]. A 50 mL centrifuge tube was filled with 1 g of powder. Subsequently, 10 mL of distilled water was added to the tube, and the solution was heated at 80°C for a duration of 30 min. After cooling, the solution was centrifuged at 2,200 rpm for 15 min. The solubility and swelling powers of the powder were calculated using the following equations:

(10) $$\begin{array}{c} \text{Solubility}=\frac{\text{weight of dried sample in the supernatant}}{\text{weight of the original sample }}\times 100, \end{array}$$

(11) $$\begin{array}{c} \text{Swelling power}=\frac{\text{weight of the paste }}{\text{weight of the dry matter }}\times 100. \end{array}$$

### 2.6. Oil Absorption Capacity

Oil absorption capacity was determined as per the method of Brishti et al. [29] with slight modification. One gram of powder was dispersed in 10 ml of oil and thoroughly stirred for 60 min and then centrifuged at 2,200 rpm for 20 min which resulted in the separation of the oil from the mixture. Further, oil was separated using a pipette, and the weight was measured. The amount of oil absorbed by the powder gave the measure of oil absorption capacity.

### 2.7. Thermal Properties (Volumetric Specific Heat, Thermal Diffusivity, and Thermal Conductivity)

Thermal properties were measured using the Hot Disk TPS 500 S instrument, manufactured by Hot Disk AB in Göteborg, Sweden, at NIFTEM-K in Sonipat, Haryana, India. The Hot Disk (HD) operates based on the transient plane source method, as originally described by Gustafsson [30]. In this study, the powder samples were placed inside a hollow cylinder and analyzed using Kapton sensors (Model-5465F2) with a radius of 3.2 mm. The measurements were conducted at room temperature, and repeatability was ensured by performing the measurements ten times. The thermal conductivity values were reported in units of Wm^−1^ K^−1^, the thermal diffusivity values were expressed in units of ×10^−6^ m^2^s^−1^, and the volumetric specific heat values were given in units of MJm^−3^ K^−1^.

### 2.8. Statistical Analysis

The wild banana seed powder was analyzed for all the properties at five different moisture contents (6.16%–19.56% db). The collected data were statistically analyzed by applying one-way ANOVA using Minitab 18 software, with a significance level set at *p* < 0.05. Additionally, linear regression analysis was performed to establish the regression equation and coefficient of determination (*R*^2^) for all the parameters.

## 3. Results and Discussion

### 3.1. Bulk Density, Tapped Density, and Porosity of Porous Powder

**Figure 2:**
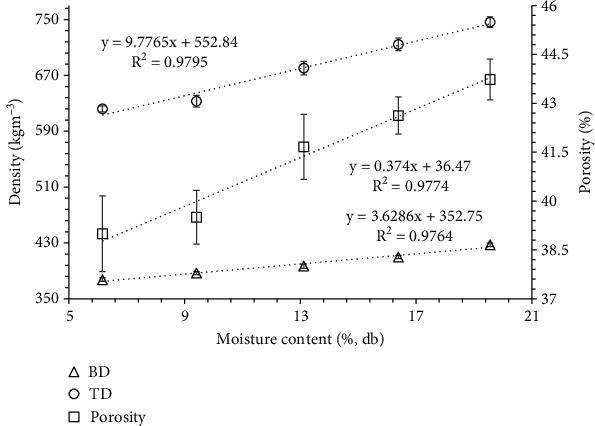
Bulk density, tapped density, and porosity of wild banana seed powder as a function of moisture.

Bulk density, tapped density, and porosity showed a significant (*p* < 0.05) increase with an increase in the moisture content (6.16-19.56% db) of banana seed powder. As shown in Figure 2, both bulk and tapped density showed a linear increase, ranging from 377.37 to 427.36 kg m^−3^ and 622.08 to 746.33 kg m^−3^, respectively, with the increment in moisture content from 6.16% to 19.56% db. Also, the porosity of banana powder showed a linear increment from 38.99 to 43.74% with an increase in moisture content. The relationship of bulk density, tapped density, and porosity with moisture content are expressed in

(12) $$\begin{array}{c} \rho_{\text{b}}=3.6286\text{Mc}+352.75\;\left(R^{2}=0.9764\right), \end{array}$$

(13) $$\begin{array}{c} \;\rho_{\text{t}}=9.7765\text{Mc}+552.84\;\left(R^{2}=0.9795\right), \end{array}$$

(14) $$\begin{array}{c} \varepsilon =0.374\text{Mc}+36.47\;\left(R^{2}=0.9774\right). \end{array}$$

The increase in both bulk and tapped densities can be attributed to the greater weight gain of the powder compared to its volume expansion as the moisture content increases [18]. The bulk density of proso millet, barnyard millet, finger millet, kodo millet, foxtail millet, and little millet flours exhibited similar increasing trends as the moisture content increased from 8.63% to 28.21% db [31]. Also, Balasubramanian et al. [32] conducted an experiment involving the grinding of black paper using a micropulverizer with different screen aperture sizes (0.5 mm, 1.0 mm, and 1.5 mm), varying feed rates (8 kg/h, 16 kg/h, and 24 kg/h), and constant rotor speed (3000 rpm), while increased the moisture content from 5.5% to 17.6% (db). The results showed that bulk density (536.7-1425 kg m^−3^) and tapped density (490-1760 kg m^−3^) of powder increased with an increase in moisture content. Similarly, Barnwal et al. [33] observed an increase in bulk density (622.1-635.3 kg m^−3^) and porosity of black pepper powder with an increase in moisture content from 4 to 8% wb. In addition, Siliveru et al. [34] found a positive correlation (*R*^2^ = 0.96) between tapped density of hard red spring, hard red winter, hard white winter, soft red winter, and soft white winter wheat flours and moisture content (10-14% wb). This could be because the particles occupied interparticulate void spaces, and the collapse of mechanical interlocking during tapping could have reduced the volume of the samples. Recently, Li et al. [35] found an increasing trend in the bulk density of *γ*-alumina and pseudo boehmite powder with weight percentages of added water of 0, 10, 25, 40, and 50%. In contrast, Mohite et al. [24] observed contrasting trends in the bulk density and tapped density of tamarind seed powder (avg. particle size 0.39–0.45 mm) produced by a hammer mill. The study found that as the moisture content increased from 5 to 15% db, both the bulk density (484 to 384 kg/m^3^) and tapped density (648–534 kg/m^3^) decreased. In addition, Probst et al. [36] reported that the bulk and tapped densities of corn and corncob powder, produced using a hammer mill, exhibited a decreasing trend as the moisture content increased. This indicates that the density of a powder not only relies on its moisture content but also depends on other factors such as particle size and shape.

Porosity quantifies the void spaces within a material by representing the ratio of the volume of voids to the total volume, typically ranging between 0% and 100%. Studies have reported similar increasing trends in porosity with an increase in moisture content, attributed to a decrease in the ratio of bulk and true density for jamun seeds [13], black pepper powder [33], tiger nuts [37], and cocoa beans [38]. In contrast, Kumar et al. [11] reported a decrease in porosity of banana seeds with an increase in moisture content. However, the information available on the porosity of the flours with moisture content is limited.

### 3.2. Hausner's Ratio and Carr's Compressibility Index

Hr and CI significantly (*p* < 0.05) increased with an increase in moisture content (6.16-19.56% db) of banana seed powder. Hr and CI values ranged from 1.59 to 1.75 and 37.07 to 42.74%, respectively, with the increment in moisture content from 6.16% to 19.56% db. The relationship of Hr and CI with moisture content is expressed in

(15) $$\begin{array}{c} \text{Hr}=0.3886\text{Mc}+35.096\;\left(R^{2}=0.89\right), \end{array}$$

(16) $$\begin{array}{c} \text{CI}=0.0109\text{Mc}+1.532\;\left(R^{2}=0.90\right). \end{array}$$

Geldart and Wong [39] devised a discrimination method for Hr, categorizing powders into different groups based on their Hr values: powders with Hr < 1.25 were classified into groups A, B, or D, those with Hr values ranging from 1.25 to 1.4 exhibited semicohesive properties, powders with Hr values between 1.4 and 2.0 were considered cohesive, and powders with Hr > 2.0 were classified as hardened. In this study, Hr falls in the range of 1.4-2.0 at a given range of moisture content indicating the cohesive characteristics of banana seed powder. Furthermore, Cui et al. [40] mentioned that if the CI value is above 40%, it shows cohesive characteristics, and a CI value below 40% is classified as hardened. In this study, the CI value was above 40% at moisture content of 13.12%, 16.39%, and 19.56% db, indicating cohesive characteristics of banana seed powder. However, at moisture content of 6.16% and 9.41% db, CI value was below 40%, indicating hardened characteristics of banana seed powder.

Particle size enlargement decreases compressibility [41]; conversely, an escalation in moisture content leads to increased compressibility due to the development of interparticle bonds through cohesion [42, 43]. Furthermore, contrasting results were observed for milk-malted millet powders, and the Hr and CI were observed to decrease as moisture content increased, indicating an improvement in flow [44]. It could be due to the adsorption of moisture by powder particles can cause them to adhere to one another, forming agglomerates through the influence of liquid-liquid bridges and interparticle forces. Thereby, this process ultimately reduces the cumulative surface area of contact between the particles, resulting in improved flowability of the powders [45]. This general trend was not observed for banana seed powder with increment in moisture from 6.16 to 19.56% db. Similarly, Probst et al. [36] noted no significant change in the compressibility index and Hausner's ratio of grounded corn and corncob powder with increasing in moisture content ranging from 10.39 to 20.13% wb. Powders with low values of CI and Hr demonstrate better flowability, whereas higher values indicate poor flow properties of the powder [24].

### 3.3. Angle of Repose, Spatula, Fall, and Static Coefficient of Friction

**Figure 3:**
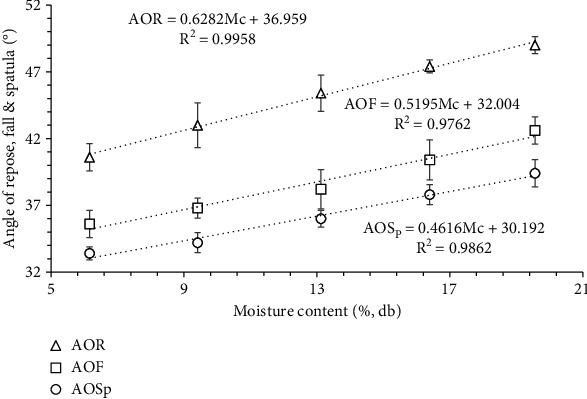
Variation in the angle of repose, angle of fall, and angle of spatula as a function of moisture content.

The angle of repose (AOR), angle of spatula (AOSp), and angle of fall (AOF) significantly (*p* < 0.05) increased with an increase in moisture content (Figure 3). The angle of repose, angle of spatula, and angle of fall exhibited a linear increase, ranging from 40.6° to 49°, 33.4° to 39.4°, and 35.6° to 42.6°, respectively, with the increment in moisture content from 6.16% to 19.56% db. The variation in the AOR, AOSp, and AOF with moisture content is expressed in

(17) $$\begin{array}{c} \text{AOR}=0.6282\text{Mc}+36.959\;\left(R^{2}=0.9958\right), \end{array}$$

(18) $$\begin{array}{c} \text{AOSp}=0.4616\text{Mc}+30.192\;\left(R^{2}=0.9862\right), \end{array}$$

(19) $$\begin{array}{c} \text{AOF}=0.5195\text{Mc}+32.004\;\left(R^{2}=0.9762\right). \end{array}$$

These measurements are important in industries that handle powders, such as pharmaceuticals, food, and chemical industries. They help to determine the flowability and handling characteristics of powders, which can affect the efficiency and quality of the manufacturing process [46]. Particularly, the angle of repose is an indirect measure of powder flow and indicates the flowability of the powders [47]. The angle of spatula is a measure of the angle of internal friction powder's cohesiveness and indicates the flowability of the powders. In addition, it is a measure of the angle at which a powder will remain stationary on a flat surface when a spatula is inserted into it and then slowly withdrawn. The angle of fall is a measure of the angle between the slope of the collapsed powder pile and the horizontal plane [25].

Similar increasing trend of AOR as banana seed powder was observed by Mohite et al. [24] for tamarind seeds grounded by hammer mill (23.74-26.56°) and attrition mill (32.61-34.99°) in the range of moisture content 5-15% db, respectively. In contrast, other researchers have reported decreasing trends of the angle of repose for basundi mix [45], milk-malted millet powders [44], and ice-cream powder [48] with an increase in moisture content ranging from 3 to 9% db. This might be the formation of the larger particles with agglomeration due to an increase in the Mc which caused a decrease in the interparticular forces among the particles causing the rolling of the powder particle over each other and resulting decrease in the AOR of powder. However, in the case of wild banana powder, increased interparticle attraction with increased moisture content could have shown the opposite results leading to an increase in AOR value. Furthermore, banana seed powder showed higher AOR values as compared to banana seeds [11]. Carr Jr [49] suggested that an AOR below 30° indicates good flowability, 30°–45° some cohesiveness, 45°–55° true cohesiveness, and >55° sluggish or very high cohesiveness and very limited flowability. In this study, at Mc range 6.65-10% db, AORs fall in the range of 30°–45° indicating some cohesive characteristics of banana seed powder, and in the Mc range of 10-20% db, AORs fall in the range of 45°–55° indicating the true cohesiveness of banana seed powder.

Furthermore, Ganesan et al. [50] studied the flow properties of distiller dried grains with different levels of solubility as a function of moisture content (10-30% db). The results indicate that the AOR (42.13-45.13°), AOF (35.40-37.77°), and AOSp (55.92-61.50°) increased with an increase in moisture content. For the banana seed powder, the angle of spatula value is less than 40° so it can be considered under the category of freely flowable powder for the given range of moisture content in this study.

**Figure 4:**
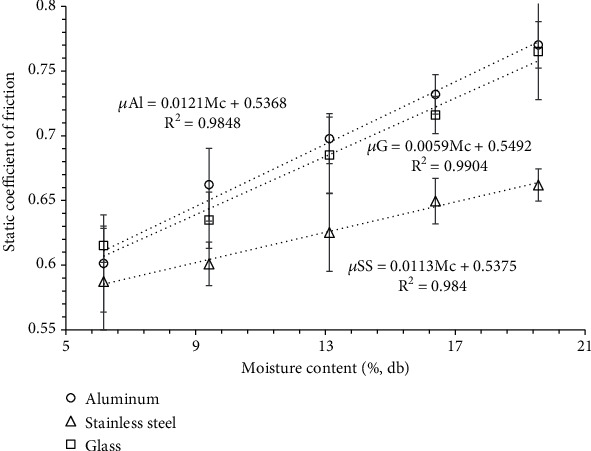
Variation in the angel of slide for different moisture contents over different surfaces.

The static coefficient of friction (*μ*) of wild banana seeds on three surfaces (aluminium, stainless steel, and glass) with different moisture contents between 6.16% and 19.56% (db) is presented in Figure 4. *μ* varied significantly (*p* < 0.05) for aluminium, glass, and stainless steel surface with an increase in the moisture content. The *μ* was found to increase linearly from 0.58 to 0.66, 0.61 to 0.76, and 0.60 to 0.77 for stainless steel, glass, and aluminium surface, respectively, in the moisture range of 6.16%–19.56% (db). At higher moisture contents (Mc > 10), the *μ* had similar value for aluminium and glass surface. The reason for the increased *μ* at higher moisture content may be the water present in the banana seeds offering a cohesive force on the surface of contact, and the surface becomes sticky [11]. The variation in *μ* of wild banana seed powder and varying moisture content for aluminium and glass surface is expressed in

(20) $$\begin{array}{c} \mu \text{Al}=0.0121\text{Mc}+0.5368\;\left(R^{2}=0.9848\right), \end{array}$$

(21) $$\begin{array}{c} \mu \text{G}=0.0059\text{Mc}+0.5492\;\left(R^{2}=0.9904\right). \end{array}$$

Barnwal et al. [33] conducted a study on the engineering properties of cryogenic and ambient ground black pepper, examining their behavior in relation to moisture content ranging from 4 to 10% w.b. The findings revealed that in the case of cryogenic ground black pepper, the static coefficient of friction (*μ*) exhibited a linear increase from 0.525 to 0.969 for plywood surfaces and from 0.206 to 0.613 for galvanized iron surfaces. Similarly, for ambient ground black pepper, *μ* demonstrated a similar increasing trend, ranging from 0.421 to 0.929 for plywood surfaces and from 0.176 to 0.593 for galvanized iron surfaces. However, no significant changes in *μ* were observed for the mild steel surface when exposed to either cryogenic or ambient ground black pepper. Similarly, other researchers have reported increasing trends of the static coefficient of friction of alfalfa grind for plexiglass (0.315-0.493), galvanized sheet (0.356-0.528), rubber (0.629-0.836), polished steel (0.269-0.289) surface [51], locust bean seed for plywood, galvanized iron, aluminium, stainless steel surfaces [52], and coffee powder for glass, paper board, and thermocol surfaces [53] with an increase in moisture content ranging from 7 to 28% db.

### 3.4. Water Activity, Solubility, Swelling Power, and Oil Absorption Capacity

**Figure 5:**
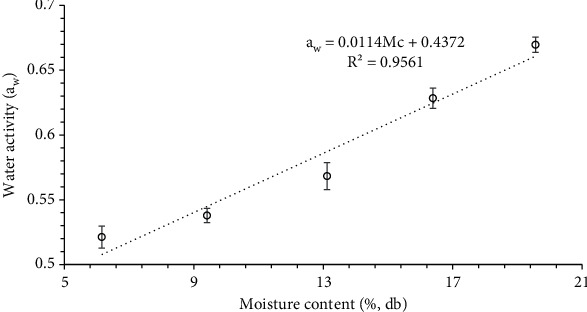
Water activity as a function of moisture content.

**Figure 6:**
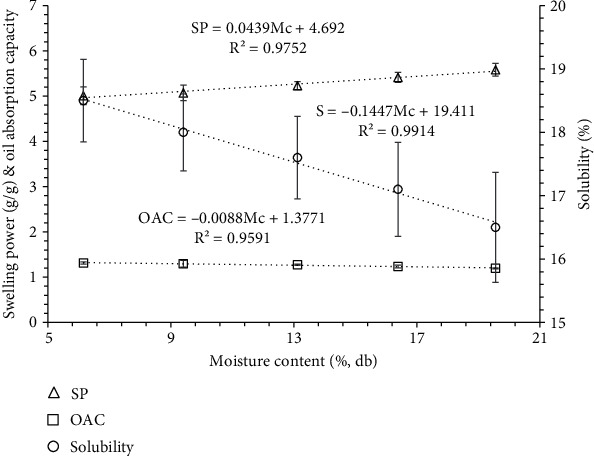
Variation in the solubility, swelling power (SP), and oil absorption capacity (OAC) at varied moisture content.

Water activity and swelling power of banana seed powder significantly (*p* < 0.05) increased with an increase in moisture content (Figures 5 and 6). The water activity and swelling power exhibited a linear increase, ranging from 0.52 to 0.67 and 5.0 g/g-5.58 g/g, respectively, with the increment in moisture content from 6.16% to 19.56% db. The increased adsorption of the moisture by the banana seed powder is responsible for the increase in the water activity of the sample as the Mc of the sample was increased. Swelling power is an important parameter to access the quality, and it is linked with protein and starch composition.

Solubility (18.50-16.50) and oil absorption capacity (1.31-1.19) of banana seed powder significantly (*p* < 0.05) decreased with an increase in moisture content in the given range (Figure 6). Solubility is a very important parameter to access the powder characteristics in an aqueous solution and represents digestibility. OAC depends on the hydrophobic nature of the powder, particle size, and overall charge density [54]. The variation in water activity (*a*_w_), swelling power (SP), solubility (*S*), and oil absorption capacity (OAC) with moisture content was expressed in

(22) $$\begin{array}{c} a_{\text{w}}=0.0114\text{Mc}+0.4372\;\left(R^{2}=0.9561\right), \end{array}$$

(23) $$\begin{array}{c} \text{SP}=0.0439\text{Mc}+4.692\;\left(R^{2}=0.9752\right), \end{array}$$

(24) $$\begin{array}{c} S=-0.1447\text{Mc}+19.411\;\left(R^{2}=0.9914\right), \end{array}$$

(25) $$\begin{array}{c} \text{OAC}=-0.0088\text{Mc}+1.3771\;\left(R^{2}=0.9591\right). \end{array}$$

A study conducted on milk powder revealed a decrease in solubility as the moisture content increased from 4 to 5%. The suggested possible reason for this phenomenon was the degradation of fat and protein molecules present in the powder samples, leading to the insolubility of these constituents as the moisture content increased [55].

### 3.5. Thermal Properties (Thermal Conductivity (*λ*), Thermal Diffusivity (*α*), and Volumetric Specific Heat (*ρ*cp))

**Figure 7:**
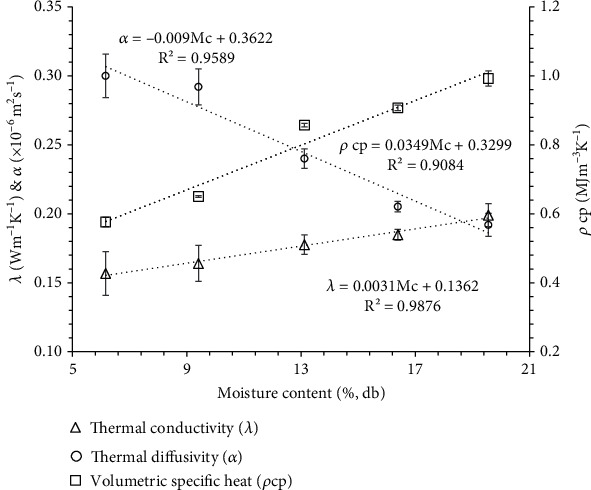
Relationship between thermal conductivity, thermal diffusivity, and volumetric specific heat at different moisture contents of wild banana seed powder.

The volumetric specific heat is a crucial property for determining the energy needed to alter the temperature of a product. On the other hand, thermal conductivity and diffusivity are two parameters that play a role in estimating the rate of heat transfer and are essential for designing processes and equipment.  Figure 7 illustrates the trends observed in *λ*, *α*, and *ρ*cp at different moisture contents ranging from 6.16 to 19.56% (db). *λ* and *ρ*cp were increased linearly from 0.16 to 0.20 Wm^−1^ K^−1^ and 0.58 to 0.99 MJm^−3^ K^−1^, respectively, whereas *α* decreased from 0.31 to 0.19 (×10^−6^ m^2^ s^−1^) with increase in moisture content from 6.16% to 19.56% (db). Similar trends of thermal properties were observed for banana seeds as a function of moisture content [11]. The variation in thermal conductivity (*λ*), thermal diffusivity (*α*), and volumetric specific heat (*ρ*cp) with moisture content is expressed by the following equations:

(26) $$\begin{array}{c} \lambda =0.0031\text{Mc}+0.1362\;\left(R^{2}=0.9876\right), \end{array}$$

(27) $$\begin{array}{c} \alpha =-0.009\text{Mc}+0.3622\;\left(R^{2}=0.9589\right), \end{array}$$

(28) $$\begin{array}{c} \rho \text{cp}=0.0349\text{Mc}+0.3299\;\left(R^{2}=0.9084\right). \end{array}$$

Mahapatra et al. [56] found a similar increasing trend of thermal conductivity (0.057-0.073 Wm^−1^ K^−1^) and decreasing trend of thermal diffusivity (0.122-0.106 mm^2^ s^−1^) of rice flour powder at different moisture contents ranging from 5.2 to 16.7% (db). Similarly, other researchers have reported similar trends of the thermal properties for borage seeds [57], velvet bean [58], Ekpoma rice flour [59], ice-cream powder [48], and three varieties of fennel seeds [60] with increasing moisture content. With the increase in moisture content and water being a good conductor of heat, there was a rise in the thermal conductivity of banana seed powder. Conversely, the decrease in thermal diffusivity could be attributed to the overall increase in material density resulting from the higher moisture content in the banana seed powder.

## 4. Conclusion

In conclusion, this study discusses the significant impact of moisture content on the various engineering properties of wild banana seed powder. An increase in the moisture content led to a significant increase in the bulk density, compact density, and porosity, with the Hausner ratio (HR) indicating cohesive characteristics. Furthermore, the angles of repose, spatula, and fall exhibited linear increase with rising moisture levels. The static coefficient of friction was found to vary depending on the surface material. Water activity and swelling power increased significantly, while solubility and oil absorption capacity decreased as moisture content increased. Thermal characteristics, including thermal conductivity and volumetric specific heat, exhibited an upward trend with higher moisture content, whereas thermal diffusivity declined. These findings underscore the significance of considering moisture content during processing, handling, and transportation of banana seed powder. Additionally, this research provides valuable insights for optimizing processes and maximizing the utilization of banana seed powder. This versatile ingredient is well-suited for various applications, encompassing bakery items, nutritional beverages, snacks, and convenience foods. Its adaptability extends to a diverse range of plant-based, nutritious products, even including formulations for baby food. Future research will focus on the development of wild banana seed powder-based food products.

## Data Availability

Data is available on request.
